# Extended Sustained Release of Propranolol Hydrochloride from Siloxane-Poly(propylene Oxide) Hybrid Material: A Multistep Mechanism

**DOI:** 10.1021/acsomega.6c00982

**Published:** 2026-04-30

**Authors:** Ranielle de Oliveira Silva, Karim Dahmouche, Celso V. Santilli

**Affiliations:** † 28340 São Paulo State University (UNESP), Institute of Chemistry, Araraquara, São Paulo 14800-060, Brazil; ‡ 28125 Federal University of Rio de Janeiro, Campus de Duque de Caxias, Duque de Caxias, Rio de Janeiro 25240-005, Brazil

## Abstract

An organic–inorganic hybrid composed of poly­(propylene oxide) (PPO) polymer chains cross-linked by siloxane nodes was selected as an amphiphilic hybrid carrier for the prolonged delivery of propranolol hydrochloride. Rubbery hybrid bodies were prepared according to a hydrolytic sol–gel route, without the use of catalysts. Efficient network formation was achieved by means of siloxane linkages, while preserving the integrity of the drug, as confirmed by ^29^Si NMR. The coupling of the hydrophobic character of the polymer with the hydrophilic nature of the siloxane nanoparticles and the urea groups located at the polymer chain extremities enabled prolonged drug release, extending up to two months. A shift from near-continuous delivery to a stair-type profile was observed when the drug loading was increased from 5 to 30 wt %. This was due to a significant increase in the fraction of drug crystallites dispersed between the hydrophobic polymer chains, as revealed by XRD, DSC, and TGA measurements, with XRD and DSC analyses also showing that penetration of water into the hybrid matrix was very slow. This caused the formation of a wet surface and a dry core within the material, leading to release according to several non-Fickian mechanisms, involving only the drug crystallites in the first steps of the process. Small-angle X-ray scattering (SAXS) analysis revealed nanoscale structural variations associated with oscillatory polymer chain expansion and contraction during the release. These pump-like changes, together with the morphological evolution of the surface and core of the hybrid material, confirmed a slow process of dissolution–diffusion of the embedded drug crystals. This generated a self-refilling reservoir effect, which maintained a steady drug flux at the interface, with the mechanisms of anomalous transport and Fickian diffusion allowing the release of the drug molecules located in the more hydrophilic nanodomains of the hybrid network after longer times. This extended the therapeutic availability of the drug, without burst release or external refilling.

## Graphical abstract

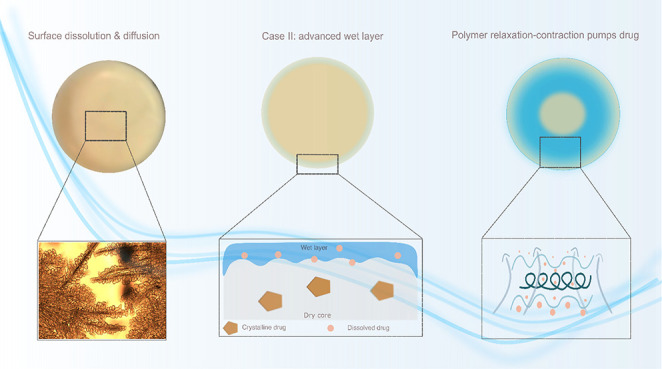

## 1 Introduction

Since the early research in the field of drug delivery, over 50 years ago, significant advances have been made by combining interdisciplinary knowledge from the areas of chemistry, biology, biomedicine, pharmacy, and engineering.^1,2^ It is now widely recognized that controlled release technologies offer many advantages over conventional drug administration methods, especially for diagnostic purposes and localized treatments, by maximizing therapeutic performance, while minimizing the risk of toxicity.^3,4^ Over the past two decades, polymer-based materials have been applied for controlled release in many biological contexts, due to their ability to carry large amounts of drugs and actively participate in the release processes in the human body, without causing adverse effects.^5–8^ Important recent advances in the development of such materials have been reported, such as on-demand stimuli-responsive delivery devices that are able to release active agents by responding to stimuli that may be exogenous (acoustic, electric, magnetic, and electromagnetic) or endogenous (pH and enzymes).^9^ Several systems of polymer-based nanoparticles capable of selectively delivering drugs to cancer cells have been developed, which reduce damage to healthy tissues.^10^ A particularly interesting technique used in the development of controlled drug delivery systems is electrospinning, which can produce polymeric biomaterials able to mimic the extracellular matrix and provide a cell-friendly environment.^11,12^ A multifunctional coating consisting of trilayer electrospun fibers that could spatially compartmentalize and sequentially release three different drugs was recently developed,^13^ offering a promising therapeutic strategy for postoperative complications of orthopedic implant surgery.

However, there are few reported studies concerning the development of biocompatible or biodegradable polymer-based materials for long-term release of drugs, because most of these systems present poor chemical and dimensional stability, with low resistance to degradation over longer periods. This particularly affects the development of new therapies employing highly water-soluble drugs, which tend to dissolve rapidly and are often eliminated in the body before acting effectively,^14^ so it is essential to develop long-lasting delivery systems suitable for drugs with fast clearance. An example of a fast-absorbing drug is propranolol hydrochloride (1-(1-methylethylamino)-3-(1-naphthyloxy)-propan-2-ol hydrochloride), a nonselective β1 and β2 adrenergic blocker prescribed for heart and cardiorespiratory problems such as arrhythmia, angina, and high blood pressure.^15^ According to the Biopharmaceutics Classification System (BCS), propranolol hydrochloride (PHC) belongs to the category of Class 1 drugs presenting high lipophilicity and permeability, resulting in 90% of the drug being rapidly absorbed and metabolized, with only 25% reaching the circulatory system.^16,17^ As a consequence, to be effective, administration of the drug necessitates 4 doses of 40 mg daily, with patients frequently requiring treatment during their entire lives.

Several different systems have recently been developed for the purpose of maintaining the delivery of PHC at therapeutic levels over an extended period.^18–23^ The presence of an amine group in its molecular structure results in propranolol behaving as a cationic drug, under physiological conditions. This ionic nature enables chemical interactions^24^ with polymeric matrices such as cellulose derivatives,^25^ chitosan,^26^ natural and semisynthetic polymers,^27^ and lipid nanoparticles.^18,21,22^ Ionic conjugation allows these materials to act as effective carriers for propranolol delivery, providing controlled release for up to 24 h. This represents an improvement over traditional drug delivery systems,^28,29^ but is still insufficient for significant enhancement of patient wellbeing.

Some recent examples of successful strategies for local drug delivery^30^ include the use of mucoadhesive devices,^31^ intranasal administration,^32^ transdermal systems,^33^ and 3D-printed devices.^34,35^ The last technique was shown by Wu et al.^35^ to provide excellent local release performance, with 80% of the drug being released through the porous matrix within 10 days. However, these systems generally exhibit low drug-loading efficiency,^36^ with the release profiles primarily governed by the solubility of the gelatinous matrix.^30,34^ On the other hand, several studies in the last 20 years have evidenced that organic–inorganic hybrid materials (OIHMs) represent an attractive alternative to purely organic or inorganic systems for biomedical applications, especially drug delivery.^37^ In these nanocomposites, inorganic and (bio)­organic phases are interpenetrated at the nanometer scale, leading to unique mechanical, electrical, thermal, optical, and biological properties. The evolution of OIHM synthesis processes during the last years has led to the development of multifunctional and multistructured hierarchical systems exhibiting biocompatibility and bioefficiency.^38,39^

Sol–gel chemistry,^40^ in particular, has led to improvements of OIHMs in the biological field.^38^ This process is based on synthesis routes in liquid solution, at ambient or moderate temperatures, allowing the incorporation of molecules with low thermal stability, such as biological substances and drugs.^41^ Hybrid organic–inorganic materials obtained by means of sol–gel strategies frequently employ silica or siloxane-based networks as the inorganic component.^42^ These inorganic phases provide multifunctional cross-linking nodes that enhance mechanical stability, transparency, and chemical resistance, while the polymeric phase contributes to flexibility and processability. The rigid siloxane nodes dispersed within the polymeric matrix generate a heterogeneous environment at the nanoscale, which influences molecular mobility and transport processes.^43^ Such structural organization enables the tuning of physicochemical properties by adjustment of the organic–inorganic ratio and network connectivity, allowing control of solvent interactions, cross-linking density, and diffusion pathways. As a result, siloxane-based hybrid systems have been widely explored as versatile platforms for biomedical applications including drug delivery systems and advanced biomaterials.^44^

Especially attractive for drug delivery are the siloxane-polyether OIHMs, composed of poly­(oxyethylene) (PEO) or poly­(oxypropylene) (PPO) chains cross-linked by siloxane nanoparticles, forming a flexible three-dimensional network.^45^ Molina et al.^46^ showed that using these materials, often called “ureasils” due to the presence of urea groups connecting the siloxane nodes and the polymer chains,^47^ it is possible to control water penetration and swelling, and the consequent drug release, by adjusting the ratio between the hydrophilic PEO and the hydrophobic PPO. These hybrids also present superior chemical and thermal stability, compared to most purely organic polymers, and form transparent, biocompatible, and adhesive films within minutes,^48^ broadening their pharmaceutical potential. However, their capacity for high drug loading and sustained release over extended periods remains unproven. Furthermore, the development of controlled drug delivery systems requires a clear understanding of the physicochemical and kinetic processes governing release, prior to *in vivo* evaluation. Although the delivery of propranolol from biomaterial-based platforms has shown therapeutic potential, detailed *in vitro* investigations correlating the release kinetics mechanism with the physicochemical evolution of the carrier matrix, under controlled release conditions, remain relatively scarce.

Moreover, classical frameworks describing drug release from polymer matrices, such as those proposed by Higuchi,^49–51^ Peppas and Ritger,^52–54^ Colombo,^55,56^ and Siepmann,^57,58^ highlight that mass transport in dense matrices may involve the coexistence of several mechanisms, including diffusion, drug dissolution, polymer relaxation, and the progressive advancement of hydration fronts.^56,59^ However, experimental validation of these mechanisms across distinct kinetic regimes remains challenging, particularly for hybrid organic–inorganic matrices, where structural constraints and multiscale organization influence transport phenomena.^60–63^ Despite the extensive use of kinetic models to describe drug release, most studies rely primarily on mathematical fitting of release profiles, without direct experimental validation of the mechanisms governing each kinetic regime. The present work investigates how the chemical and structural features of the siloxane-PPO ureasil, which determine the local molecular organization of the nanoscale structure, enable both high drug loading and continuous release of propranolol hydrochloride (PHC) for up to ∼1000 h. By combining kinetic modeling with complementary structural and thermal characterization techniques, this work provides experimental evidence supporting the occurrence of the different regimes in the release profiles. Based on this multiscale experimental approach, the findings introduce, for the first time, the concept of a self-refilling matrix, where conformational changes in the PPO chains grafted onto the siloxane nodes, triggered by the dissolution of precipitated PHC, activate the transport of the dissolved drug toward the surrounding medium. This process enables the extended delivery of this highly water-soluble drug without external refilling, maintaining therapeutic levels for weeks. The ability to sustain propranolol release for several weeks opens new routes in the design of medical biodevices.

## 2 Experimental Section

### 2.1 Synthesis of Hybrid Materials

The OIH precursor was prepared according to a standard procedure,^45^ employing PPO with molecular weight of 4000 g/mol. The reagents (3-isocyanatopropyl)­triethoxysilane (CAS No. 24801-88-5) and poly­(propylene glycol) bis­(2-aminopropyl ether) (CAS No. 9046-10-0) were mixed at a molar ratio of 2:1 and stirred in tetrahydrofuran (THF) for 12 h, under reflux. The reagents were used as received and the THF was dried prior to use. After the reaction, the THF was evaporated, yielding the hybrid precursor. The hydrolysis and polycondensation of the hybrid precursor were initiated by adding 0.4 mL of water (molar ratio [H_2_O]/[Si] = 100) to a solution containing 0.5 g of precursor and 1 mL of ethanol (99%, analytical grade). Propranolol hydrochloride (PHC, lot 13ES027, purity 98%, kindly provided by Fundação Oswaldo Cruz, Brazil) was then added and the solution was magnetically stirred for 2 h, followed by ultrasonication at 320 W for 90 min. Two different PHC contents were used, corresponding to 5 and 30 wt % of the precursor. The sealed flask containing the solution was stored in an oven at 50 °C during 5 days, for formation of the hybrid gel network. After gelation, the flask was opened and the wet gel was allowed to dry at 50 °C for 5 days. The drug-free sample was named UPPO4000, while the OIH samples containing 5 and 30 wt % of PHC were named UPPO4000-5 and UPPO4000-30, respectively. High-purity water from a Milli-Q system was used in the hydrolysis step. No catalyst was added during the hydrolysis process. Measurements of pH showed that the hybrid solution without the drug had a pH of 6.8. The sample containing 5 wt % of propranolol hydrochloride had pH between 4 and 5, while the sample containing 30 wt % of propranolol hydrochloride had pH between 3 and 4.

### 2.2 Structural and Thermal Characterization

Solid-state ^29^Si magic angle spinning nuclear magnetic resonance (MAS/NMR) spectra were acquired at 25 °C, using a spectrometer (Inova, Varian) operating at 300 MHz, at the Larmor frequency of 59.59 MHz. The spectra were obtained from Fourier transform of the free induction decays following a single π/2 excitation, with a dead time repetition rate of 3.2 s. Chemical shifts are reported relative to a tetramethylsilane reference standard. Proton decoupling was always used during acquisition of the spectra and the uncertainty in the chemical shift values was less than 0.2 ppm.

Fourier transform infrared (FTIR) measurements employed a spectrometer (VERTEX 70, Bruker) equipped with an attenuated total reflectance (ATR) accessory. The spectra were acquired between 4000 and 400 cm^–1^, with accumulation of 64 scans and resolution of 2 cm^–1^.

Wide-angle X-ray scattering (WAXS) measurements were performed at the Brazilian National Synchrotron Light Laboratory (LNLS, Campinas, Brazil). The beamline was equipped with an asymmetrically cut and bent silicon (111) monochromator that produced a monochromatic (λ = 0.154 nm) and horizontally focused beam. A charge-coupled device (CCD) detector was positioned at 250 mm from the sample. These conditions enabled experiments to be performed in the 2θ range between 0.8° and 27°.

Small-angle X-ray scattering (SAXS) measurements were also performed at LNLS. A position-sensitive X-ray detector (PILATUS 300k) was used to record the SAXS intensity, *I*(*q*), as a function of the modulus of the scattering vector *q* (*q* = (4π/λ)­sin­(θ/2), where θ is the scattering angle). Each SAXS pattern corresponded to a data collection time of 9 s. The detector/sample distance was set at 616 mm, which allowed a scattering vector range between 0.1 and 4.8 nm^–1^. The scattering intensity was normalized by subtracting the background scattering and the natural intensity decay of the incident beam.

Thermogravimetric analysis (TGA) was performed with heating of 20 mg of the sample from 25 to 500 °C, at 5 °C min^–1^, under a constant flow of nitrogen (50 mL min^–1^) (SDT Q600 analyzer, TA Instruments). Differential scanning calorimetry (DSC) measurements were performed in the temperature range from −100 to 300 °C, with 10 mg of sample and a heating rate of 10 °C min^–1^ (SDT Q100 analyzer, TA Instruments). The samples were cooled and heated under a constant flow of nitrogen (50 mL min^–1^).

### 2.3 In Vitro Drug Release

The PHC release was quantified by UV–vis spectroscopy (Cary 50, Varian), at λ = 290 nm, with construction of an analytical calibration curve. Disk-shaped samples (18 mm diameter and 3 mm thickness; see Figure 1) were prepared, allowing the application of mathematical models for unidimensional drug release. Samples containing 5 and 30 wt % PHC were immersed in 0.5 and 1 L volumes of ultrapure Milli-Q water, respectively, at 35–37 °C. To ensure physiological relevance and enable meaningful interpretation of the release kinetics, the *in vitro* drug release experiments were performed at 37 °C. This temperature corresponds to standard *in vitro* conditions used to simulate the human body environment, as recommended in the U.S. Food and Drug Administration guidelines for dissolution testing.^64^

**Figure 1:**
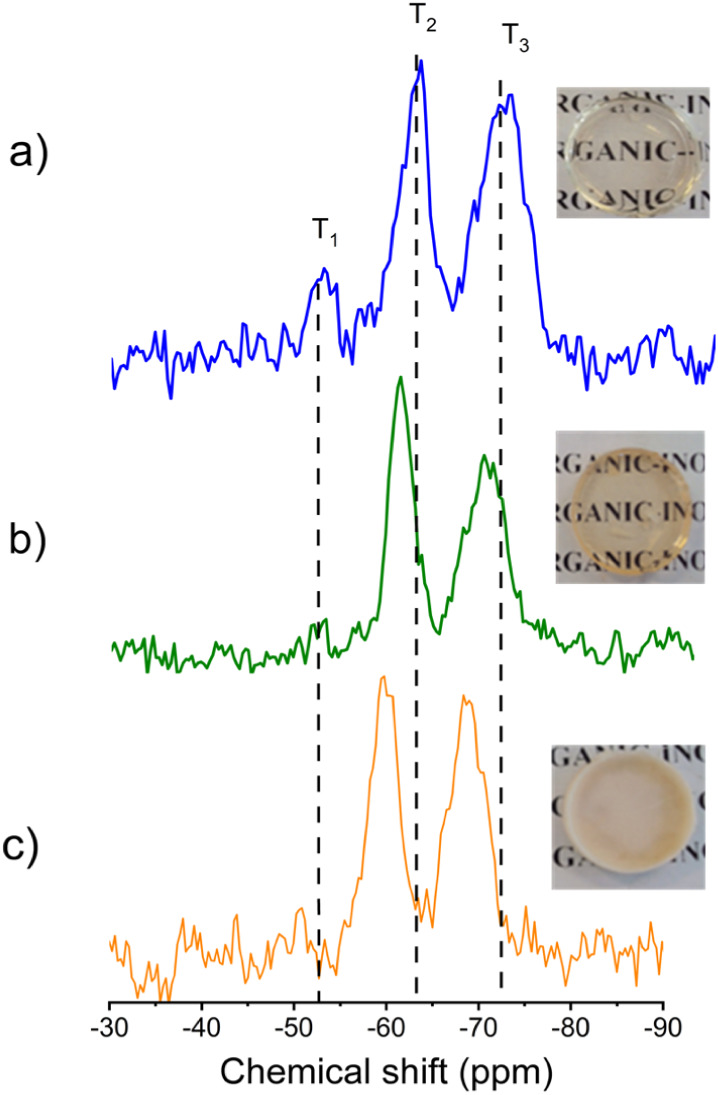
^29^Si NMR spectra and corresponding images of (a) UPPO4000, (b) UPPO4000-5, and (c) UPPO4000-30.

Considering the high aqueous solubility of PHC (∼360 mg mL^–1^),^65^ the dissolution volumes were chosen to maintain the drug concentrations within the linear range of the calibration curve and above the detection limit of the method, while avoiding saturation of the medium. This ensured *sink* conditions, prevented UV–vis signal saturation, and allowed accurate monitoring of both the initial burst release and the subsequent sustained release phases.^17^ The relatively large volumes also assisted homogeneous mixing and minimized local supersaturation, which is particularly important for solid hydrophobic matrix systems. The release vessel was sealed to prevent evaporation. At predetermined intervals, 5 mL volumes of the medium were withdrawn for analysis and were immediately returned to the vessel, to maintain a constant volume. For each sample (containing 5 and 30 wt % PHC), the drug release experiments were performed in triplicate in three independent experiments, to ensure reproducibility. During the drug release process, the surface and bulk regions were characterized by cutting slices of superficial and internal portions of the same sample, which were submitted to analysis by the WAXS, TGA, and SAXS techniques. For each technique, this procedure was performed at specified time intervals and was interrupted when there were no further discernible differences between the two regions.

## 3 Results and Discussion

### 3.1 Local Structure and Thermal Characteristics of Drug-Loaded OIH

The ^29^Si CP/MAS NMR spectra of the pristine UPPO4000 hybrid and the drug-loaded samples (Figure 1) exhibited the three characteristic signals of the siloxane network, at −50 ppm (T^1^), −58 ppm (T^2^), and −67 ppm (T^3^), corresponding to O–Si units bonded to 1, 2, and 3 Si atoms, respectively. The degree of polycondensation (PC) was calculated by integrating the area (%) under each Si species peak and considering that the PC values expected for *T* ^1^, *T* ^2^, and *T* ^3^ were 33.3%, 66.7%, and 100%, respectively. PC increased from 81% for the pristine hybrid to 83% and 85% after drug loading (Table 1). These high values indicated that the absence of a catalyst in the sol–gel synthesis did not compromise network formation. Notably, the incorporation of PHC was accompanied by an increase in the proportion of fully condensed *T* ^3^ sites, suggesting that the drug promoted further condensation of the silica domains and enhanced the cross-linking density of the hybrid network. This is the first report of UPPO4000 hybrids for drug delivery synthesized without catalysts, yielding organic–inorganic matrices free from impurities, which is a feature of particular interest when high purity is required to minimize potential drug interactions.

**Table 1:** Degree of Polycondensation (PC) of Silicon Environments Determined from the *T*,^1^ *T*,^2^ and *T* ^3^ Contributions Obtained from ^29^Si NMR Spectroscopy

	*T* ^1^ (%)	*T* ^2^ (%)	*T* ^3^ (%)	PC (%)
UPPO4000	7	41	52	81
UPPO4000-5	5	43	52	83
UPPO4000-30	3	44	53	85

Complementary information concerning the bonding structures of the drug-loaded UPPO4000 hybrids was obtained from comparison of the FTIR spectra for the pristine hybrid matrix, propranolol hydrochloride, and the drug-loaded samples, presented in the Supporting Information (Figure S1). The spectrum for the hybrid exhibited characteristic PPO bands, including a C–H stretching band at 2970–2860 cm^–1^ and an intense C–O–C stretching band at around 1090–1020 cm^–1^, together with contributions from Si–O–Si vibrations in the same region. Signals related to the urea linkages connecting the PPO chains to the siloxane network could also be identified, particularly N–H stretching in the 3300–3200 cm^–1^ region and C=O stretching near 1630 cm^–1^. However, these bands were relatively weak, due to the low relative concentration of urea groups that acted as linkers between the PPO chains and the Si–O–Si network, as well as overlap with the broad O–H/N–H region and other matrix vibrations. After drug loading, additional bands associated with aromatic vibrations of propranolol were evident, particularly at around 1575 cm^–1^. However, no systematic shifts were observed in the broad O–H/N–H region (∼3300 cm^–1^) or in the carbonyl region (at around 1630 and 1560 cm^–1^). This behavior was due to strong band overlap and the dominance of PPO vibrations, which limited the sensitivity of FTIR to detect subtle hydrogen-bonding interactions in this system.

The WAXS pattern of PHC (Figure 2) exhibited a set of sharp peaks, characteristic of a monoclinic structure.^66^ The WAXS pattern of the pristine matrix showed only a broad band related to the amorphous siloxane phase, as observed previously for these hybrids.^49^ In contrast, the WAXS patterns of the UPPO4000-5 and UPPO4000-30 hybrids showed the set of peaks characteristic of PHC. The dispersion of this crystalline phase is evidenced by the photographs in Figure 1, showing the maintenance of transparency of the hybrid matrix after conjugation with 5 wt % of PHC and the opacity of the hybrid with 30 wt % of crystalline PHC.

**Figure 2:**
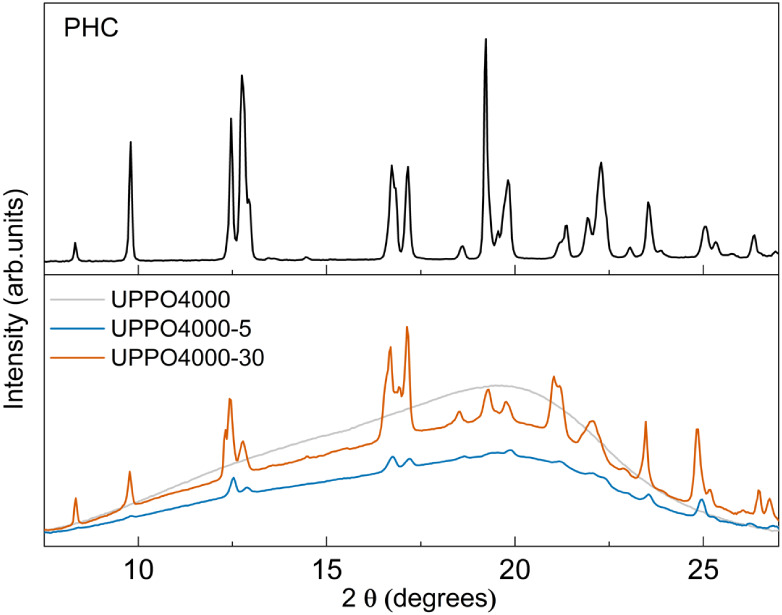
WAXS patterns of propranolol hydrochloride (PHC), the UPPO4000 hybrid matrix, UPPO4000-5, and UPPO4000-30.

Further information concerning the phases present in the PHC-loaded UPPO4000 hybrids was obtained by comparison of the DSC curves acquired during heating from −95 to 200 °C (Figure 3). The low temperature region of the DSC curves showed a sharp change of heat capacity, characteristic of the glass transition. This single glass transition and the invariance of its onset point (−67 °C, corresponding to the glass transition temperature, Tg) confirmed the absence of PHC molecules dissolved in the amorphous polymeric phase of the hybrid matrix. The absence of any exothermic event related to cold crystallization confirmed the amorphous nature of the UPPO4000 hybrid phase. The only endothermic event in the DSC curves was due to the melting of PHC at around 162 °C, with a heat of fusion of around 99 J g^–1^.^48^ For the PHC-loaded samples, the peak areas for this event corresponded to crystalline PHC fractions of ∼5% in UPPO4000-5 and ∼23% in UPPO4000-30. These low fractions of crystalline PHC, even in UPPO4000-30, together with the invariance of Tg after incorporation of the drug, indicated the dissolution of drug molecules by the hydrophilic urea groups present at the ends of the polymer chains. This coupling favored a high degree of cross-linking of the hybrid network, as indicated by ^29^Si CP/MAS NMR (Table 1). This hypothesis was supported by the WAXS pattern of the PHC-loaded hybrid with lower molecular weight (PPO of 300 g mol^–1^) and, consequently, higher urea content, where greater PHC dissolution was evidenced by the significant reduction of the crystalline drug fraction.

**Figure 3:**
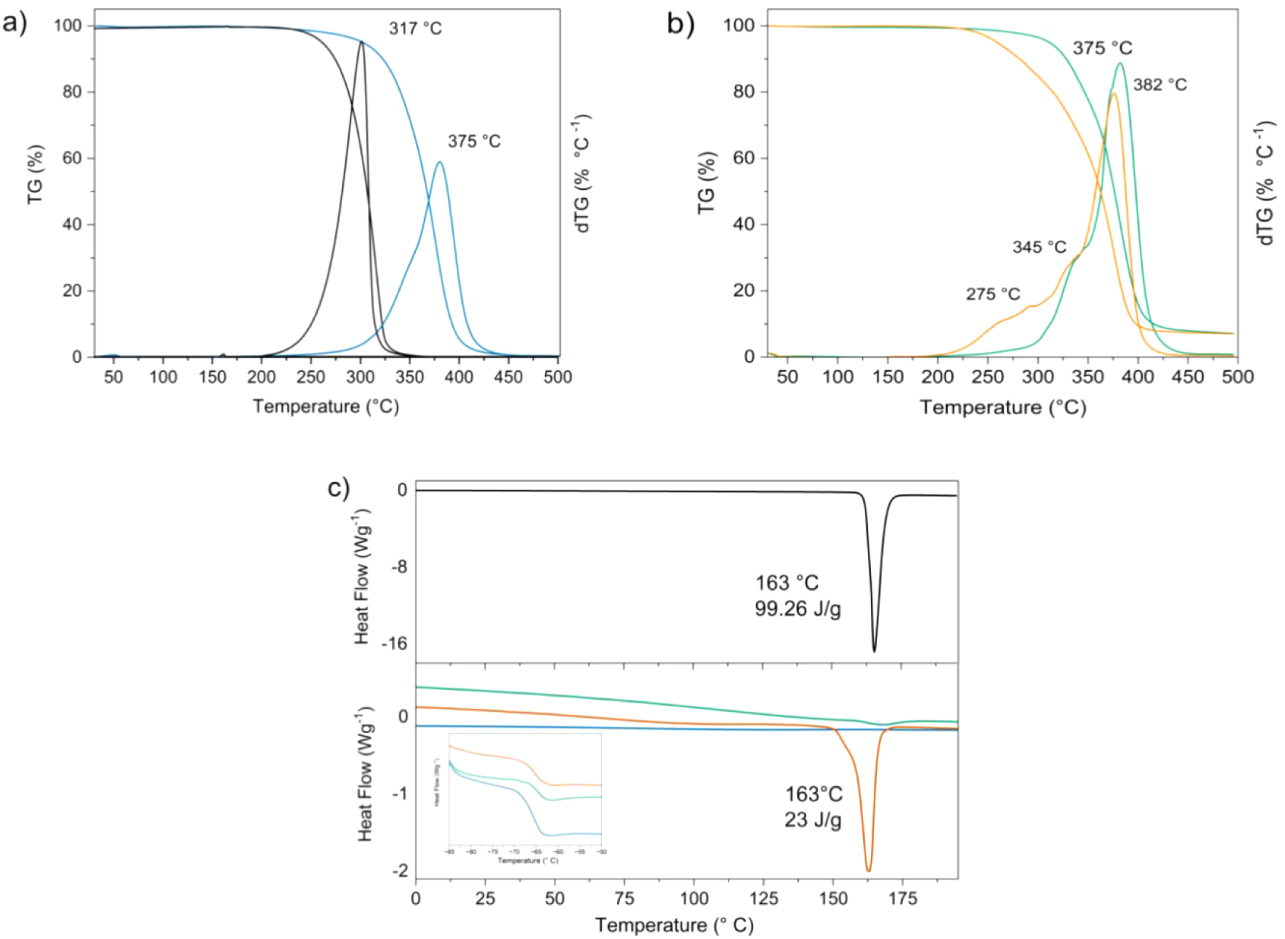
TG/dTG (a, b) and DSC (c) curves for the PHC drug (black), the UPPO4000 hybrid matrix (blue), UPPO4000-5 (green), and UPPO4000-30 (orange). The inset shows the DSC curves near the glass transition temperature.

### 3.2 Drug Release Mechanisms and Evolution of the Local Structure of the Hybrid Matrix

The cumulative PHC mass fractions released during the immersion of UPPO4000-5 and UPPO4000-30 in water (Figure 4) revealed a remarkably long-lasting release behavior. In particular, the stepped profile, characterized by a nonperiodic oscillatory slope, indicates an anomalous transport mechanism resulting from the superposition of normal diffusion with other processes such as drug dissolution and structural relaxation of the matrix.^50^

**Figure 4:**
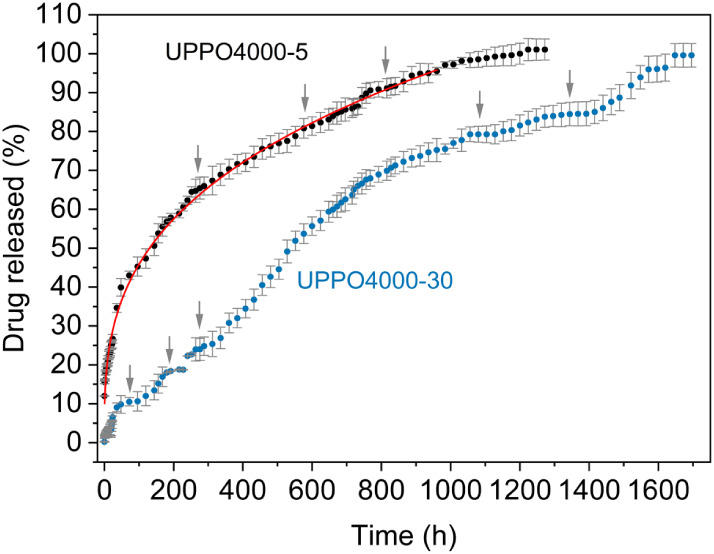
Cumulative release profiles of propranolol hydrochloride (PHC) from hybrid matrices containing 5 and 30 wt % PHC. The data are shown as mean ± SD from three independent experiments performed in triplicate.

Complex structured drug delivery systems frequently exhibit multiple kinetic regimes, due to sequential dissolution processes, diffusion barriers, or compartmentalized reservoirs, resulting in nonlinear or step-like release profiles. Similar multistage release behavior has been reported for structured polymer delivery systems, where hierarchical architectures or spatially separated domains generate distinct release events over time.^35,61,63^ More generally, drug release from polymer matrices is often governed by the simultaneous contributions of several transport mechanisms, including diffusion, matrix relaxation, and drug dissolution, which can lead to time-dependent changes in release kinetics and the emergence of multiple kinetic regimes. The models developed to describe these mechanisms have been extensively described in the classical theoretical works of Higuchi, Peppas, and Siepmann,^50,58^ and have since been experimentally confirmed for drug delivery systems with diverse compositions and structural architectures.^60,62,67^

An appropriate model describing the coexistence of different drug release mechanisms with different rates was developed by Korsmeyer and Peppas,^68^ described by the following semiempirical power law equation:

1 $$M_{t}/M_{\infty }=kt^{n}$$

where, *M*_*t*_ */M*_*∞*_ is the drug mass fraction released as a function of time *t*; *k* is a constant related to the geometric and structural characteristics of the matrix; and the value of the exponent *n* depends on the nature of the release mechanism for a determined sample geometry. It should be noted that the above model was derived from the basic equation of the Higuchi model proposed for drug release by Fickian diffusion of the molecules,^49^ but takes into consideration other mechanisms that could contribute to the release process, such as matrix degradation, swelling of the matrix in the dissolution medium, and solubility of the drug in the matrix, which also depends on the drug loading.^55^ In some cases, an instantaneous release of the drug (burst effect) is observed immediately after immersion of the matrix in the dissolution medium, with eq 1 becoming:^69^

2 $$M_{t}/M_{\infty }=kt^{n}+b$$

where *b* is the drug fraction released at *t* = 0.

The Korsmeyer-Peppas model is appropriate for analysis of the siloxane-PPO hybrid system, since it does not impose restrictive assumptions regarding matrix hydrophilicity, swelling behavior, or pore structure. Instead, the empirical exponent (*n*) allows the identification of transport regimes including Fickian diffusion, anomalous transport, and relaxation-controlled release, with applicability for hydrophobic and nondegradable and/or inert matrices. This flexibility makes the model particularly suitable for heterogeneous hybrid matrices, such as the organic–inorganic network investigated here, where diffusion, matrix relaxation, and structural reorganization, together with drug dissolution and mass transport, may contribute simultaneously to the overall drug release behavior.

A more pronounced step-like release profile was observed for the formulation containing 30 wt % PHC, indicating the existence of multiple kinetic regimes. Therefore, segmented fitting according to the semiempirical approach was used to describe the different stages of the release process. The transition points between regimes were used to define characteristic time intervals that were further investigated using complementary techniques sensitive to structural and thermal changes, including thermal analysis, XRD, and SAXS. This provided additional information to correlate the kinetic modeling with experimental observations of structural evolution within the matrix, thereby supporting the mechanistic interpretation of the release process and validating the selection of this model.

As shown in Figure 4, the release from UPPO4000–5 exhibited a burst effect, followed by a low power regime (continuous line), averaging the oscillation highlighted by the arrows. The *b* value of 12% revealed that a significant fraction of the drug molecules located at the surface of the hybrid sample was instantaneously released by contact with the aqueous medium. The burst effect was probably driven by the hydrophilic nature of PHC aggregates present on the external surface of UPPO4000.^70^ Such segregation could occur during gel aging, where water migration related to polycondensation reactions after gelation and consequent extension of the solid network carried dissolved drug to the external surface, leading to recrystallization upon evaporation during drying.

Up to 1050 h of immersion of the UPPO4000-5 sample, reasonable agreement was obtained between the experimental release curve and the theoretical function (continuous line) described by eq 2. The kinetic parameters related to the release mechanism were determined from the best fits obtained using the least-squares method, resulting in *k* = 10.5 h^–1^, *n* = 0.3, and *R* ^*2*^ = 0.99. This experimental *n* value was lower than the expected value (*n* = 0.45) for drug release from disk-shaped nonswellable hydrophobic matrices, such as UPPO4000-5, involving only a Fickian diffusion mechanism.^52^ In this process, the dissolution of drug crystallites or small drug aggregates present in the water-filled pores of the matrix is very fast and occurs when the drug is loaded at a concentration level below the solubility limit of the drug in water, which was the case for this sample (the solubility limit of PHC in water is 360 mg mL^–1^ at 37 °C).^71^ Another factor favoring the occurrence of this mechanism in the case of UPPO4000-5 was that at ambient temperature, the polymer network was well above its glass transition temperature, as shown by DSC, so the high mobility of the polymer chains favored transport of the drug molecules by diffusion.^72^ Despite these conditions favorable to diffusional transport, the lower exponent value (*n* = 0.3) indicated that the PHC release was governed by a pseudo-Fickian diffusion process.

The disparity of the *n* value, compared to the expected value for a pure Fickian diffusion mechanism, could be attributed to several factors. It is established that diffusion of drug molecules in nonswellable matrices occurs through water-filled connected pores or water-filled free volume regions of the polymer.^52^ In addition, pure Fickian diffusion is favored when there is rapid penetration of the dissolution medium in the polymer core.^72^ Since penetration of water in the bulk of the nonporous amphiphilic UPPO4000-5 sample was likely to have been very slow and nonhomogeneous, this would have hindered the access of water molecules to the crystallized PHC molecules dispersed within the bulk of the hybrid matrix. Furthermore, it has been demonstrated that the classical kinetic models,^73^ such as the Korsmeyer-Peppas model, are unable to perfectly describe drug delivery for complex and multicomponent materials, where several driving forces contribute to the release. This is the case for UPPO4000 hybrids that contain hydrophilic urea and silanol groups dispersed in a hydrophobic PPO matrix. Furthermore, eq 1 is theoretically applicable to the first 60% fraction of release from thin samples, for which the assumption of one-dimensional diffusion under *sink* conditions is valid.^52^ However, in practice, this model has been applied even when such conditions are not confirmed.^52^ To the best of our knowledge, the present work is the first to report such a very long period of release for PHC. A previous study found that even for hydrophobic matrices loaded with this drug, complete release occurred within the first 24 h.^34^ Elsewhere, sustained release during 7 days was reported for PHC incorporated in a 3D scaffold device.^35^

For the hybrid loaded with a high drug concentration (UPPO4000-30), the release profile showed lower PHC release, with several well-defined steps (Figure 4). The release mechanisms involved in each step were analyzed using a least-squares fitting procedure, resulting in the continuous lines shown in Figure 5. The parameters obtained from the Korsmeyer-Peppas model are displayed near the fitting lines corresponding to the different steps. The high coefficients of determination (*R* ^*2*^ > 0.97) confirmed the consistency of the fitting procedure used to describe the different stages of the release process. After the initial burst event, corresponding to release of around 1 wt % of the PHC, a kinetic regime well fitted by eq 1 was observed in the first 20 h of sample immersion, with *k* = 1h^–1^ and *n* = 0.3 (Figure 5a). Due to the observed heterogeneity in the UPPO4000-30 sample (Figure 1c), further structural characterizations were performed by TGA (Figure 6) and WAXS (Figure 7), to compare the evolution of the surface and the bulk of the material throughout the release process, aiming to elucidate the underlying release mechanisms.

**Figure 5:**
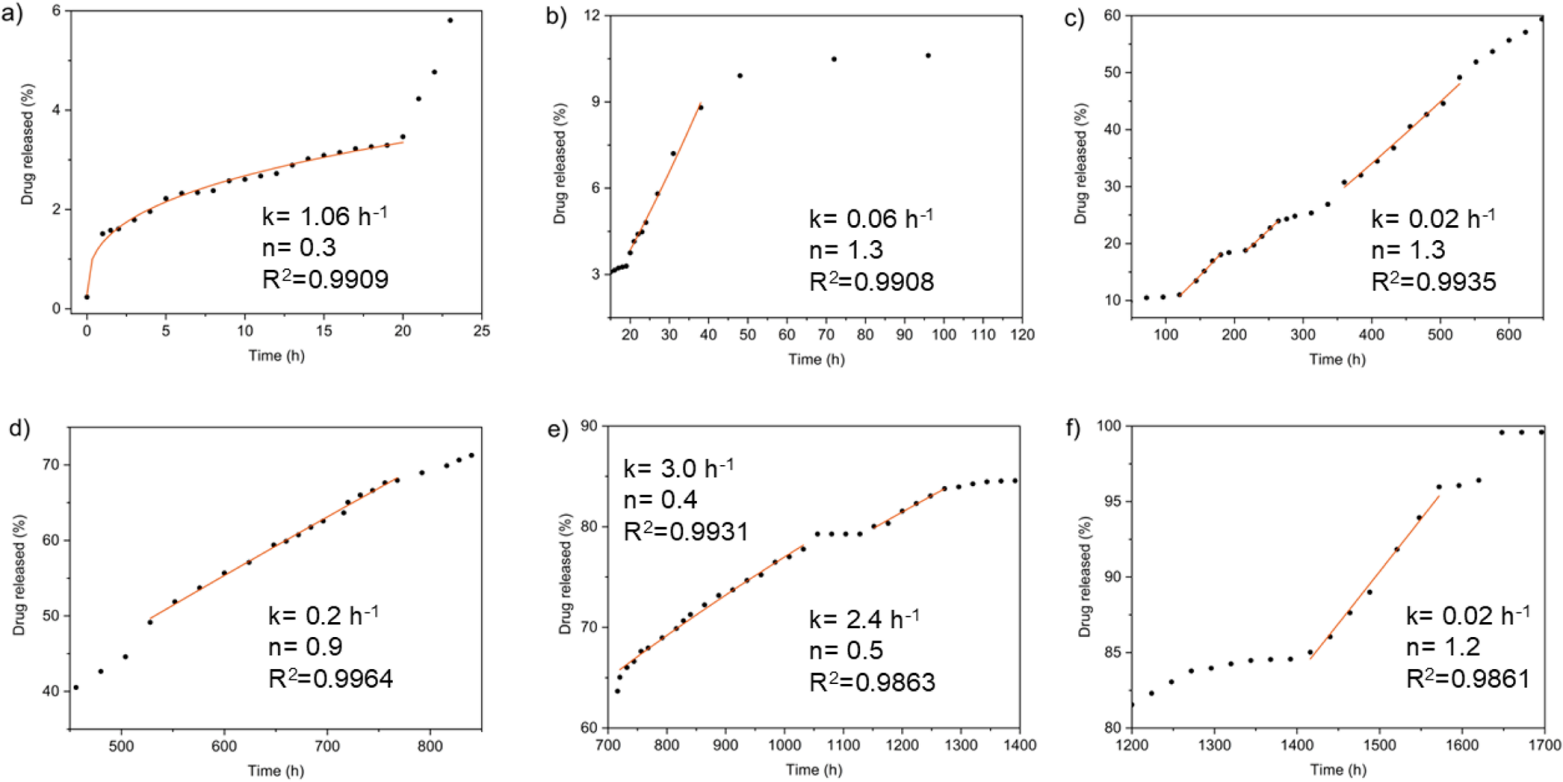
Drug release from the UPPO4000–30 sample, showing multiple release regimes: 0–20 h (a), 20–36 h (b), 120–528 h (c), 528–730 h (d), 730–1270 h (e), and 1440–1570 h (f). Each regime was individually fitted using the Korsmeyer-Peppas model. The corresponding values of the kinetic parameters (*k* and *n*) and *R^2^* are indicated in the graphs.

**Figure 6:**
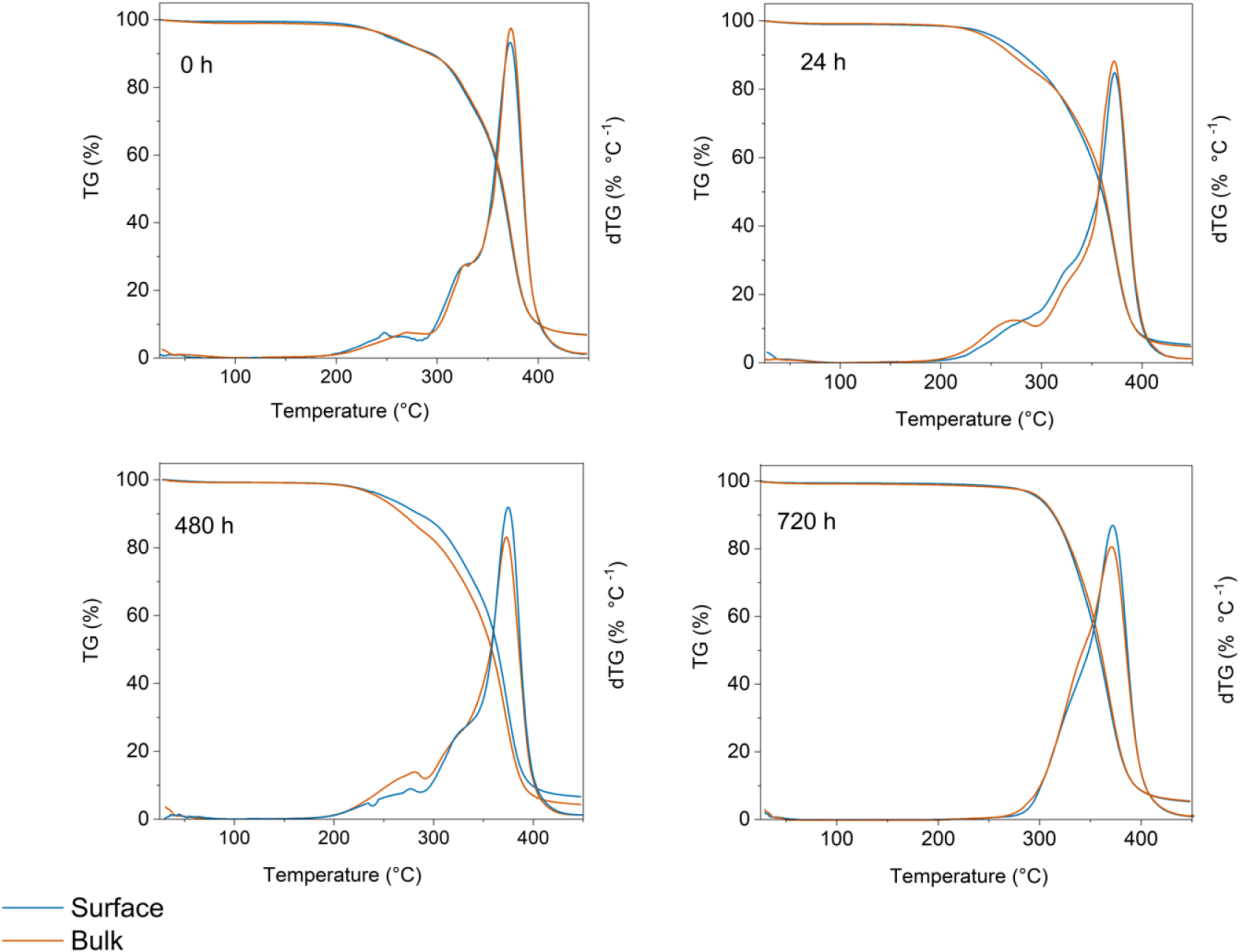
Comparison of TGA/dTG curves for the surface (blue lines) and bulk (ocher lines) of the UPPO4000-30 sample, after different drug release times.

**Figure 7:**
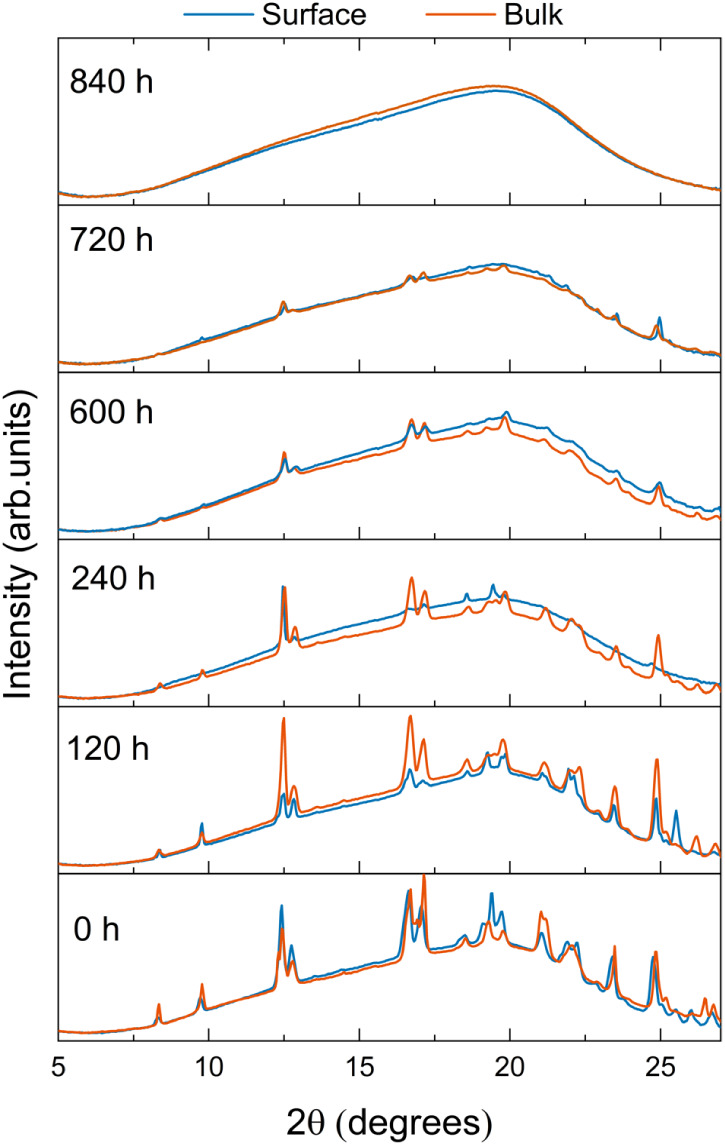
Comparison of WAXS diffractograms for the surface and bulk of the UPPO4000-30 sample after different times of drug release.

The TGA curves for the pristine PHC and UPPO4000 (Figure 3a) showed a single degradation step characterized by a single maximum of the derivative curve (dTG), at 317 and 376 °C, respectively. The PHC single maximum was preserved for UPPO4000–5, while a double maximum was observed for the UPPO4000-30 sample (Figure 3b), indicating the presence of PHC in different forms. Before the release (Figure 6, 0 h), these double maxima were similar for the surface and bulk regions of the material. After 24 h of release (Figure 6, 24 h), for the surface region, there was a less pronounced maximum of the PHC degradation rate in the lower temperature region (220–280 °C), while the TGA/dTG curves for the bulk region were not significantly affected by the release. This comparison revealed that the pseudo-Fickian diffusion mechanism for drug release during the first regime occurred at the surface region of the sample. This could be explained by the very slow penetration of water into the hybrid matrix, due to the hydrophobic nature of the PPO chains, the low mass fraction of hydrophilic urea and silanol groups, and the nonporous structure of the material. Hence, in the first release step, water molecules of the dissolution medium only penetrated a thin layer adjacent to the sample surface. For the matrix loaded with 5 wt % PHC, the burst event corresponded to approximately 12% of the total drug content, with a release exponent of *n* = 0.3 and a Higuchi constant of *k* = 10.5 h^–1^. In contrast, a much smaller burst release (1 wt %) was observed for the matrix loaded with 30 wt % PHC, with *n* = 0.3 and a substantially lower *k* value of 1.06 h^–1^, indicating a slower diffusion-driven release. This slower pseudo-Fickian diffusion could have been related to the higher fraction of drug crystallites at the surface of this sample, as indicated by the WAXS (Figure 2) and DSC (Figure 3) analyses.

The second regime shown in Figure 5b occurred between 20 and 36 h, with *n* = 1.3 and *k* = 0.06 h^–1^. For swellable or nonswellable hydrophobic matrices (regardless of their geometric shape), *n* higher than 1 usually indicates a non-Fickian release mechanism known as Super Case II.^68^ This type of mechanism is related to the difference (known as differential swelling stress) between the osmotic pressure exerted by the wet region of the sample toward the dry core and the additional stress caused by relaxation of the polymer chains at the interface between the dry and wet regions, in the direction of the wet region.^72,74,75^ This phenomenon leads to different stress components that eventually generate cracks and defects, with the consequent increase of the internal surface enhancing contact of the drug with water. However, such a release mechanism, with low *k*, is usually found for systems such as glassy polymers, where low mobility of the polymer chains hinders immediate penetration of the dissolution medium into the polymer core.^76^ This results in the formation of wet and dry regions in the sample throughout most of the drug delivery process. It should be noted that this interpretation could not be fully confirmed for the present system, because the siloxane-PPO hybrid matrix was above the glass transition temperature (Tg). Nevertheless, the hypothesis that the detected non-Fickian mechanism corresponded to Super Case II transport was consistent with the fact that it was only observed after 20 h. This time interval was sufficient for the formation of a wet region near the sample surface, which was large enough to generate osmotic pressure toward the dry core and induce Super Case II water transport (as a hydration front). Furthermore, the hybrid material exhibited similarities with a glassy polymer, due to the glassy character of the inorganic siloxane nodes, the fact that it was a dense, nonporous, and nonswellable system, and the existence of structural constraints imposed to the extremities of the polymer chains by the siloxane domains.

After this period, three other release periods presenting non-Fickian mechanisms were observed (all with *n* = 1.3 and *k* = 0.02 h^–1^), between 120 and 180 h, 220 and 260 h, and 320 and 530 h, always after a period without PHC release (Figure 5c). During periods without drug release, motion of the water penetration front toward the sample core should lead to formation of new wet slabs of increasing volume and increasing osmotic pressure, favoring the increase of differential swelling stress and, consequently, again inducing the non-Fickian mass transport event. This showed that the most important factor for drug release according to this hydration-front-controlled (HFC) mechanism was the very slow penetration of the dissolution medium inside the sample, due to the amphiphilic character of the hybrid matrix, independent of the mobility of the polymer chains. As already noted, slow penetration of water in the system was due to the nature of the affinity for water of the nonporous hybrid matrix with a large component of hydrophobic PPO chains and a small component of hydrophilic urea and silanol moieties that could be locally percolated by the propranolol chloride crystallites, which were also hydrophilic.

It was noteworthy that after 120 h of release, the WAXS pattern of the sample surface still showed several intense diffraction signals of propranolol chloride crystals (Figure 7), revealing that after the first HFC process, the region close to the surface of the hybrid matrix still contained a significant amount of drug crystallites. This was confirmed by the TGA curve obtained for the sample surface after the same period (Figure 6), which still showed a first maximum of the PHC degradation rate between 220 and 280 °C. In fact, after 120 h of immersion, only around 12% of the drug mass had been released (Figure 6c). The second and third HFC processes contributed more significantly to release of the drug crystallites located close to the hybrid surface, as shown by the WAXS patterns acquired after 240, 320, and 600 h (Figure 7).

However, the sequential HFC processes were insufficient to release the drug crystallites located in the core of the sample, or most of the drug molecules dispersed within the hybrid matrix. Evidence of these remaining species was provided by the WAXS pattern of the bulk fraction after 480 h of immersion (Figure 7) and the corresponding dTG curve (Figure 6), which still showed the characteristic PHC degradation maxima. The release of most of these species, evidenced by the WAXS patterns after 600 and 720 h (Figure 7) and by the thermograms for the same times (Figure 6), was governed by another mechanism occurring between 530 and 760 h, which presented *n* = 0.9. For disk-shaped nonswellable systems, a value of *n* between 0.45 and 1 indicates a release mechanism known as anomalous diffusion,^52^ where the release is governed partially by pure Fickian diffusion and partially by drug dissolution. Non-Fickian release, with a predominant role of drug dissolution, occurs when the drug is loaded in the matrix at a concentration level above the solubility limit of the drug in water. In this case, water molecules access drug aggregates or crystallites located in the free volume of the polymer matrix, which dissolve at their saturation concentration in water, followed by diffusion through the water in the free volume to the surrounding dissolution medium outside the polymer matrix. When release occurs by dissolution, the value of *k* is lower than for a pure diffusion release mechanism, due to the time required for the dissolution process.

This mechanism is consistent with the nature of the drug species transported during this stage: (i) dissolution and release of remaining drug crystallites according to dissolution-controlled anomalous diffusion; and (ii) Fickian diffusion of molecularly dispersed drug interacting with urea groups within the hybrid network. As expected, the value of *k* = 0.3 h^–1^ was lower than observed for the pure Fickian mechanism observed up to 20 h of release. After 530 h of sample immersion, water had probably penetrated most of the sample volume, which diminished the mechanical stresses that led to the HFC transport in the previous release stages, favoring anomalous diffusion. It should be noted that similar to HFC transport, anomalous diffusion is usually observed in glassy polymers when the temperature is below Tg.^72,76^ The occurrence of such a mechanism above Tg corroborated the very slow diffusion of water in the UPPO4000-30 sample.

The two subsequent drug delivery steps, occurring during the periods 780–1030 h and 1150–1270 h (Figure 5e), were controlled by pure Fickian diffusion, with *n* values of 0.4 and 0.5, respectively. At these advanced stages of the release process, WAXS measurements (Figure 7) revealed the almost complete disappearance of drug crystallites, indicating that the remaining drug fraction was predominantly dispersed at the molecular level within the hybrid matrix. Optical microscopy images of the partially hydrated samples (Figure S2, Supporting Information) provided visual evidence of heterogeneous hydration within the matrix during the first 3 weeks of water immersion, revealing a hydrated outer region and a less hydrated inner domain, consistent with hydration-front propagation. Under these conditions, transport was governed by Fickian diffusion of the molecularly dispersed drug through the hydrated polymer network, resulting in higher *k* values (3 and 2.4 h^–1^), compared to the dissolution-controlled regime. The existence of a period without release, between these last two diffusion regimes, further evidenced the very slow penetration of water into the hybrid matrix. Finally, after prolonged immersion, when the hydration front reached the last relatively dry region near the sample core, new mechanical stresses originating from the difference between osmotic pressure and forces related to relaxation of the polymer chains induced a new hydration-front-controlled process (*n* = 1.2 and *k* = 0.02 h^–1^), allowing release of the remaining drug fraction between 1440 and 1570 h (Figure 5f).

### 3.3 Evolution of the Nanostructures of Hybrids During the Release Process

SAXS analyses were used to investigate the evolution of the nanostructural features of UPPO4000-5 and UPPO4000-30 during the drug release process (Figures 8 and 9). Before PHC release, the SAXS patterns for both samples presented an interference peak at medium *q*-range and a decreasing regime at low *q*-range. The interference peak was due to spatial correlation between the siloxane nanoparticles located at the ends of the polymer chains,^45^ which was restricted to specific regions where the formation of larger hybrid aggregates (hybrid correlation domains, CDs) resulted in the low *q*-range scattering regime.^77,78^ The origin of this two-level hierarchical structure was related to the conformation of the polymer chains, since those inside the aggregates were entangled, while extended conformation occurred between the aggregates (Figure 10).

**Figure 8:**
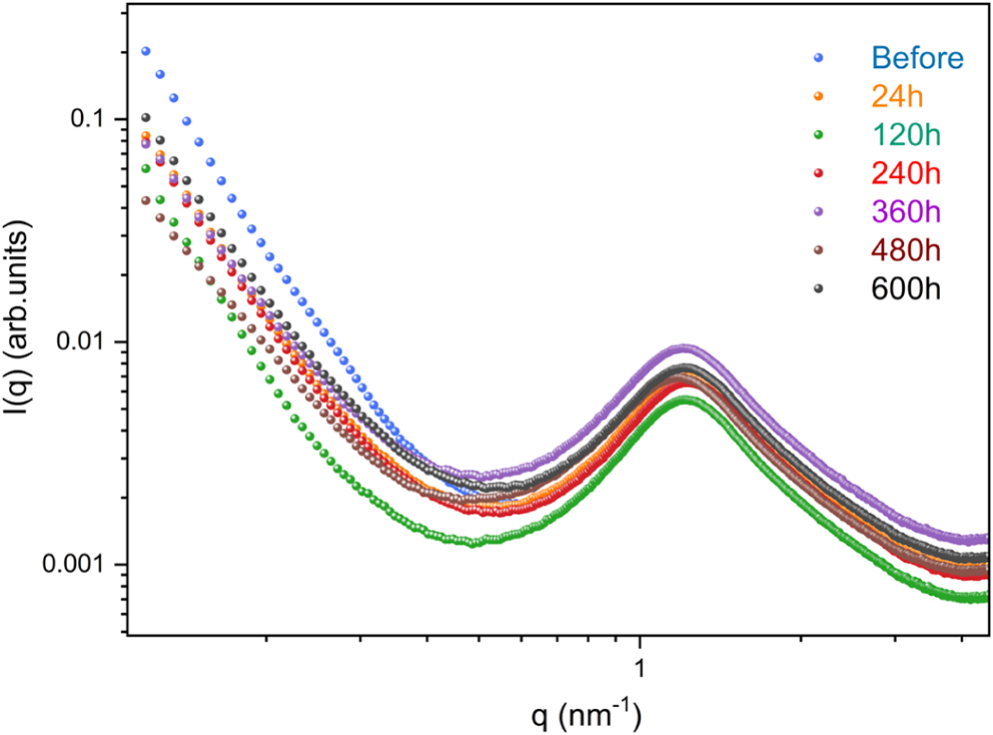
Temporal evolution of SAXS patterns during the release of propranolol hydrochloride from the UPPO4000–5 hybrid material.

**Figure 9:**
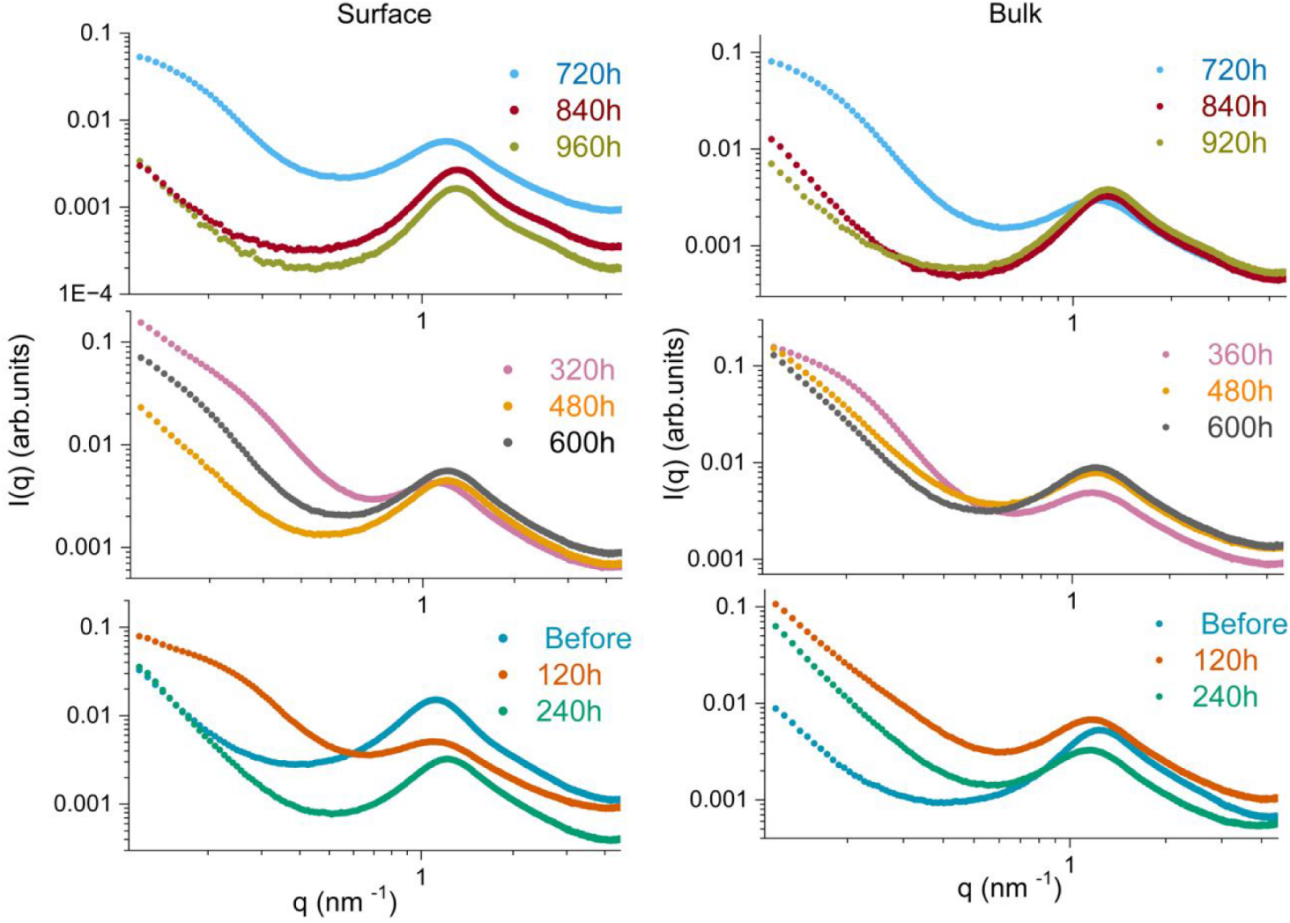
Comparison of SAXS patterns obtained for the surface and bulk of UPPO4000–30 during drug release.

**Figure 10:**
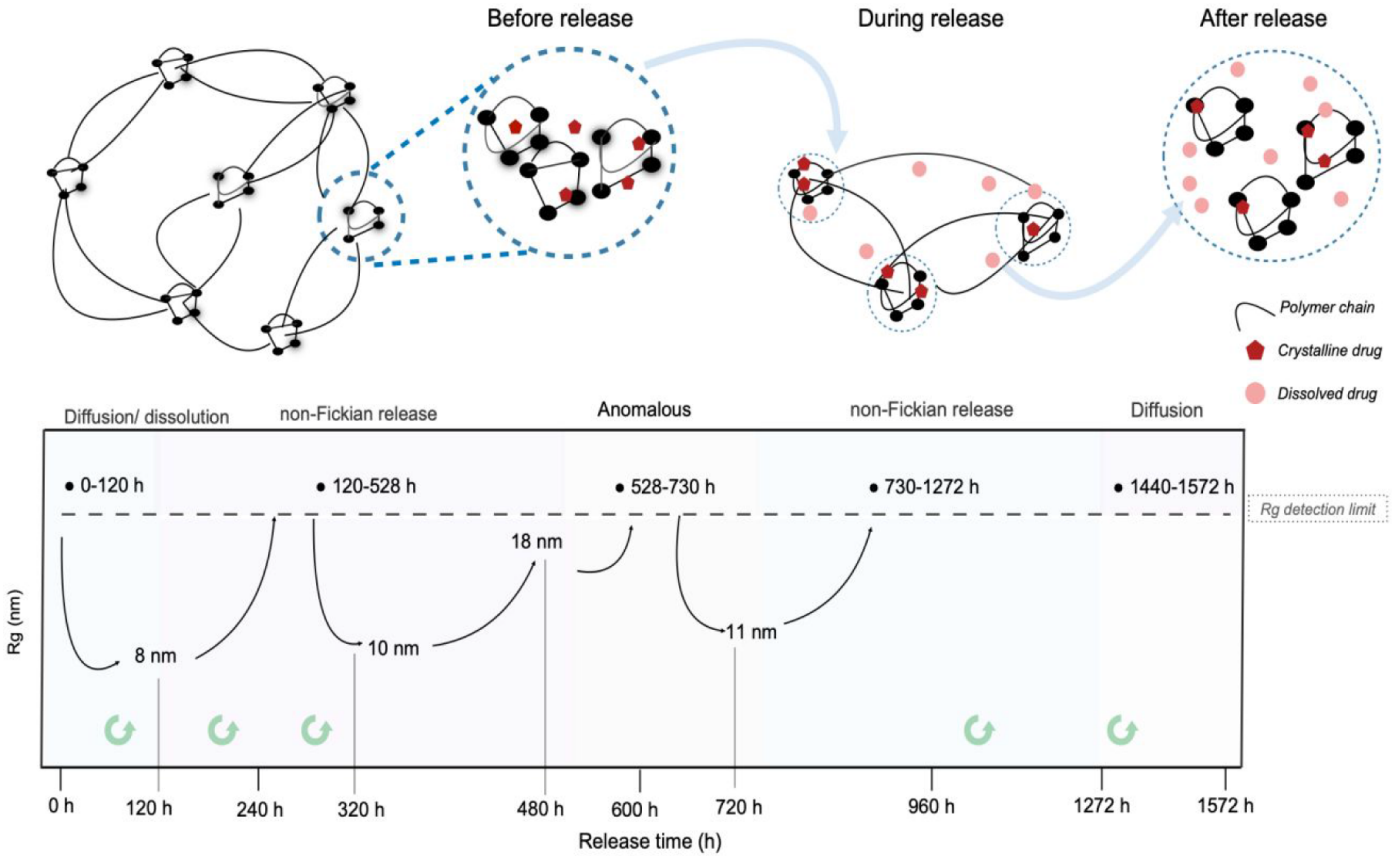
Oscillatory nanostructural temporal evolution correlated with the drug transport mechanism and the pumping release regimes. The green arrows represent the refilling time during the absence of release.

The evolution of the SAXS curves during PHC release was completely different for the two studied samples. The SAXS pattern for UPPO4000-5 showed no significant changes during drug delivery (Figure 8). In contrast, the nanostructure of UPPO4000-30 was greatly affected by the different drug delivery dynamics, as evidenced by the SAXS patterns acquired for the bulk and near-surface of the sample after different times of PHC release (Figure 9). No significant change of the *q*_max_ peak position in the SAXS curves for the UPPO4000–30 surface was observed during the first 120 h of release, with the interparticle correlation distance (*d* = 2π/*q*_max_) remaining at around 5.2 nm. However, there was a tendency toward a Guinier plateau, indicating a decrease of the average gyration radius (Rg) of the CDs during this initial period. The Rg value for the CDs after 120 h was around 8 nm, as determined from the best fitting of the SAXS curve by the theoretical Guinier equation.^79^ The decrease of the CD size, without alteration of the siloxane interparticle distance, could be explained by the disentanglement of some of the PPO chains located inside the correlation domains. During the third HFC transport regime, an increase of the CD size was evidenced from comparison of the surface SAXS patterns acquired after 120 and 240 h of release, which revealed a shift of the Guinier plateau toward a lower q-range, making it undetectable by the equipment employed in this work (Figure 9). Similar behavior was observed during the fourth HFC transport regime, comparing the patterns at 320 h (Rg = 10 nm) and 480 h. During these regimes, WAXS and TGA measurements evidenced release of the drug from the PHC crystallites located close to the sample surface. Due to the hydrophilic character of PHC and the hydrophobic nature of the PPO chains, it could reasonably be assumed that the drug crystallites were located in the hydrophobic regions of the hybrid matrix, where the polymer chains were extended, while the drug fraction dispersed at the molecular scale was associated with the more hydrophilic urea-rich aggregates. Therefore, during the HFC transport regimes, interaction of the extended hydrophobic PPO chains with the highly polar medium consisting of water molecules and an increasing number of ions resulting from dissolution of the PHC crystallites could induce contraction and shrinkage of the chains, leading to the formation of larger CDs by linkages between different smaller CDs.

Interestingly, as observed after 120 h of release, a decrease of the CD size was also observed after 320 h, as shown by the presence of the Guinier plateau (Figure 9). These behaviors occurring at the end of intervals between two HFC regimes could be explained by the less polar character of the environment around the PPO chains during these stages, due to the previous release of ions produced by dissolution of the PHC crystals, which led to a degree of disentanglement of the chains. The SAXS curves for the bulk and surface of the sample were similar after 240 and 320 h, revealing that release of some drug crystallites from the bulk material began during the third HFC step, but increased in significance at more advanced release stages, as already evidenced from the WAXS and TGA results. This was consistent with the increasing penetration of the water front toward the sample core, between two HFC regimes.

No constant step was observed between the fourth HFC regime and the anomalous diffusion regime. For the latter regime, further information could be obtained by comparison of the SAXS patterns acquired after 600 and 720 h of release. The tendency toward a Guinier plateau (Figure 9), observed for the surface after 600 h, was consistent with the previous dissolution and release of most of the PHC crystals. As expected, this tendency was less pronounced for the bulk of the material, which still contained a larger fraction of drug crystallites. An average Rg value of 18 nm was obtained for the CDs at the sample surface. After 720 h of release, the Guinier plateau was more evident and extended for both the surface and bulk of the sample, associated with a decrease of the Rg value of the CDs to 11 nm. The decrease of the CD size was exactly the opposite of the behavior observed during the HFC transport regimes. Different to the behavior during such regimes, this finding suggested that dissolution of the drug crystallites did not induce any pronounced increases in the concentrations of ions in some regions of the sample. This was consistent with the proposed transport mechanism after the fourth HFC transport stage, which ended at around 530 h, in which water penetrated most of the sample volume, favoring movement of the drug species and avoiding high local concentrations. Hence, the most significant effect on the nanostructure during the anomalous diffusion regime appeared to be the lowering of the drug molecule concentrations in the wet regions of the sample, due to Fickian diffusion, leading to a less polar environment around some of the PPO chains and inducing chain disentanglement.

The SAXS curves acquired during the subsequent release regimes controlled by Fickian diffusion mechanisms provided additional useful information concerning the penetration of water within the UPPO4000-30 hybrid. After 840 and 960 h of immersion (Figure 9), the SAXS curves for the surface and bulk of the UPPO4000-30 sample showed unexpected size increases of the hybrid CDs, evidenced by an absence of the Guinier plateau. This could be explained by the penetration of water in some drug-free regions of the sample, constituted of extended PPO chains between the CDs, leading to chain entanglement. Such behavior was consistent with the hypothesis of the diffusion of water molecules to siloxane-rich and urea-rich regions, where the drug molecules were dissolved. This contrasted with the previous stages of drug delivery, where the presence of the hydrophilic drug crystallites was the most important driving force for the progressive penetration of water into UPPO4000-30. Since the sample volume fraction occupied by the CDs was low (the Guinier plateau was associated with a diluted system of scatterers) and no more drug crystallites were present in the material, the diffusion and transport of water molecules toward the CDs implied a degree of interaction with drug-free regions of the extended polymer chains.

It should be noted that this mechanism also occurred during the last HFC regime and could explain the absence of a Guinier plateau in the SAXS pattern for the sample bulk at 1520 h (Figure 9). During this period, aqueous solution penetrated the last remaining dry region of the sample core, which did not contain drug crystallites, and the release was controlled by diffusion of the drug molecules. After complete drug release, the SAXS pattern was unchanged (Figure 9), which could be explained by the absence of dissolution of drug crystallites during this period, avoiding a pronounced increase of the concentration of ions and significant polarity changes in the liquid medium around the PPO chains.

The sequence of these modifications, involving alternating periods of swelling and contraction of the PPO chains, evidenced that the hybrid matrix acted in a manner analogous to a self-pumping device, as illustrated in Figure 10. The correlations between the nanostructure and the mass transport profile reinforced the hypotheses concerning the association between the multimodal release mechanism of the UPPO4000-30 hybrid system and the physical state and distribution of the drug within the matrix. The initial release stages were dominated by the dissolution of surface and embedded drug crystallites, which enhanced the ingress of water into the matrix. As these crystallites dissolved, the polarity and osmotic profile of the local environment changed, leading to progressive wetting and activation of previously dry and drug-free regions rich in extended PPO chains. The arrival of water in these internal regions induced chain entanglement, due to increased mobility, particularly in the absence of salt-induced polarity shifts. This conformational response acted as a polymer-driven pumping mechanism, which contributed to the self-refilling reservoir effect observed during the later stages of release. As a result, a sustained concentration gradient was maintained at the matrix-solution interface, supporting continued drug delivery even after loss of the surface crystals.

This complex interplay of morphological features, water transport, and polymer dynamics allowed the matrix to progressively release its drug load from the interior outward, until reaching complete depletion. The unchanged SAXS pattern after total release further confirmed the absence of major structural rearrangements once drug diffusion was complete and no more crystallites remained to modulate local environments. These findings highlight the crucial roles of matrix hydrophobicity, nanoscale organization, and drug loading heterogeneity in tailoring long-acting drug delivery systems. Hence, the ability to modulate delivery by means of internal structural dynamics emerges as a promising strategy for the development of high-purity, catalyst-free devices with predictable long-term performance.

## 4 Conclusions

This work evidence the potential of siloxane-PPO organic–inorganic hybrids for very long-term drug delivery, not only of propranolol chloride, but also of many other hydrophilic drugs. The very prolonged release process in this system is due to two factors. The first is the essentially hydrophobic and nonporous nature of the matrix in which the drug is embedded, which avoids fast penetration of the dissolution medium in the material. The second is the presence of hydrophilic domains (drug crystallites, siloxane, and urea) dispersed in some regions of the matrix, which attract water molecules and allow a nonhomogeneous and very progressive penetration of the dissolution medium.

A particularly novel finding was the observation of correlation between the successive drug delivery mechanisms and the periods of expansion and shrinkage of the organic–inorganic spatially correlated volume, which acted as a drug-delivery pump (a self-refilling reservoir effect). The results demonstrated that significant changes in conformation of the polymer chains could occur, even in nonswelling hydrophobic systems, with the interaction between water molecules and the drug crystallites located in the hydrophobic polymer matrix mainly occurring during the first regimes of the drug delivery process. The diffusion of water toward the more hydrophilic urea-rich aggregates essentially occurred in the last regimes, with release of the molecular drug fraction according to Fickian diffusion.

Another interesting contribution of this study is the hypothesis that the non-Fickian mechanism (hydration-front-controlled transport) detected in the first stages of drug release was Super Case II, which is usually observed for glassy polymers. The most important factor for drug release by such a mechanism, above the glass transition temperature, was found to be the slow penetration of the dissolution medium inside the sample, independent of polymer chain mobility, which opens new perspectives in the development of polymer-based hydrophobic matrices for drug delivery.

The unique combination of high drug-loading capacity and slow water penetration, associated with progressive drug dissolution and long-term release, as observed for the ureasil-PPO hybrid matrices, highlights their potential for use in implantable or coating-based therapeutic devices. In particular, the ability to sustain, for several weeks, drug delivery from a dense, nonporous, and mechanically stable material makes these hybrids promising candidates for applications such as drug-eluting coatings for medical implants, long-term subcutaneous delivery systems, or reservoir-type biomaterials for localized therapy. In addition, the structural stability of the hybrid network, combined with its adjustable hydrophilic–hydrophobic balance, further enables precise modulation of solvent penetration and mass transport, providing a versatile platform for tailoring release profiles to different application requirements. Beyond biomedical applications, such characteristics may also be attractive for the controlled release of active agents in other fields, including agriculture, for example in the sustained delivery of fertilizers or agrochemicals.

Finally, the new findings of this work provide a crucial *in vitro* kinetic foundation that complements existing *in vivo* studies concerning propranolol-loaded biomaterials. Unlike studies of these systems that primarily focus on evaluation of the biological outcomes, the present study provides a detailed mechanistic analysis of the behavior of drug release from hydrophobic polymeric matrices, including distinct release regimes, burst kinetics, and diffusion control. These results contribute to bridging the gap between laboratory-based dissolution studies and therapeutic applications, providing predictive data that can assist in the design, optimization, and regulatory validation of propranolol delivery platforms for targeted local therapy.

## Supplementary Material



## Notes

Figure S1 shows the FTIR spectra (ATR mode) of all components, including the unloaded matrix, pure propranolol hydrochloride, and samples containing 5 and 30 wt % of the drug. Figure S2 presents photographs of the samples, illustrating their visual changes over extended drug release time (PDF)

## ◆

Helmholtz-Zentrum Berlin für Materialien and Energie GmbH, Department for Electrochemical Energy Storage, 14109, Berlin, Germany
