# Supplementary material for: Extended Sustained Release of Propranolol Hydrochloride from Siloxane-Poly(propylene Oxide) Hybrid Material: A Multistep Mechanism
Source: ACS Omega. 2026 Apr 30;11(18):27168–82. doi: 10.1021/acsomega.6c00982 (PMC13176998; doi:10.1021/acsomega.6c00982)
Supplement: Supplementary file 1 [file ao6c00982_si_001.pdf]

Extended sustained release of propranolol hydrochloride from siloxane-poly(propylene oxide) hybrid material: a multiple-step mechanism

Ranielle de Oliveira Silva [a*‡], Karim Dahmouche [b], Celso V. Santilli [a*]

[a] São Paulo State University (UNESP), Institute of Chemistry, Araraquara, SP, 14800-060, Brazil

‡ Present Address: Helmholtz-Zentrum Berlin für Materialien und Energie GmbH, Department for Electrochemical Energy Storage, 14109, Berlin, Germany

[b] Federal University of Rio de Janeiro, Campus de Duque de Caxias, Duque de Caxias, RJ, 25240-005, Brazil

* Corresponding authors. E-mail addresses: ranielle.de_oliveira_silva@helmholtz-berlin.de and santilli@iq.unesp.br

Supporting information

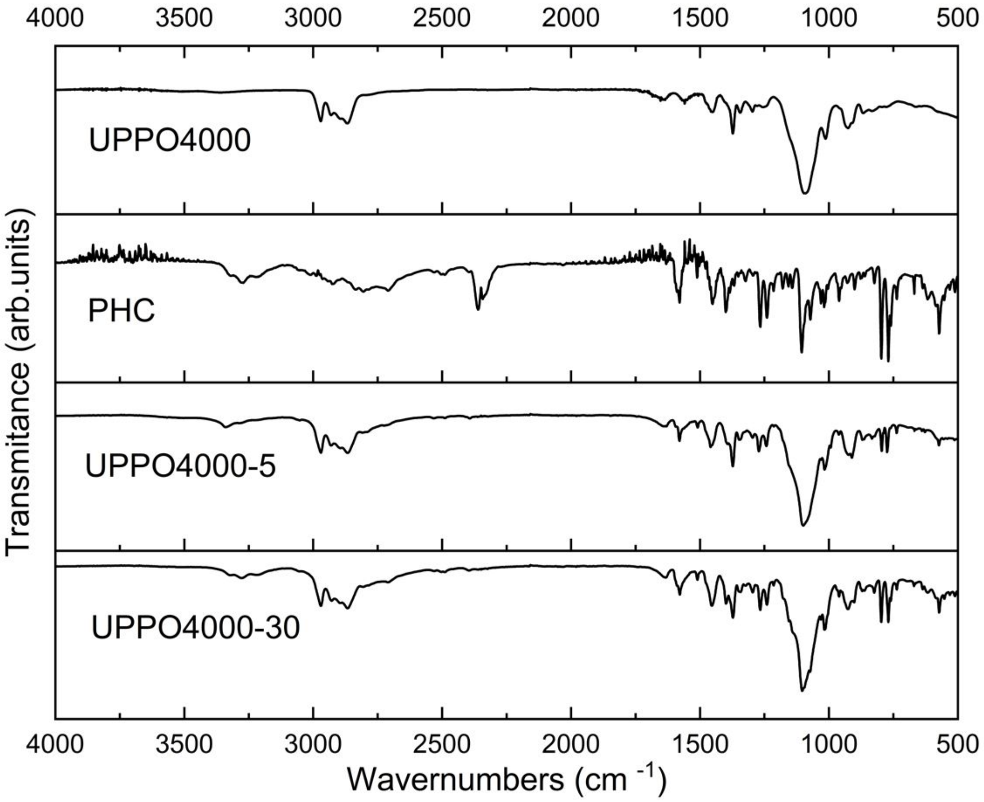

Figure S1: ATR-FTIR spectra of the blank siloxane–PPO hybrid, pure propranolol hydrochloride, and hybrid materials loaded with 5 wt% and 30 wt% of drug, used to evaluate possible interactions between the matrix and the pharmaceutical compound.

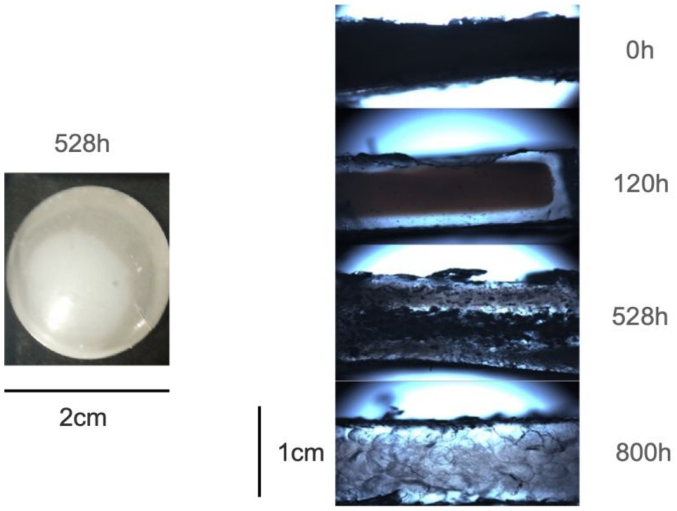

Figure S2: Photographic images and optical microscopy and showing heterogeneous hydration of the ureasil–PPO matrix after partial exposure to the dissolution medium. The images reveal visually distinguishable hydrated outer regions and a less hydrated inner domain, indicating the development of hydration fronts during solvent penetration.
